# Effects of Kinesio taping on forearm supination/pronation performance fatigability

**DOI:** 10.1186/s12891-022-05068-4

**Published:** 2022-02-09

**Authors:** Chih-Kun Hsiao, Yi-Jung Tsai, Chih-Wei Lu, Jen-Chou Hsiung, Hao-Yuan Hsiao, Yung-Chuan Chen, Yuan-Kun Tu

**Affiliations:** 1 Department of Orthopedics, E-Da Hospital, No. 1 E-Da Rd, Yuan-Chau District, Kaohsiung, Taiwan; 2 Department of Industrial and Systems Engineering, Chung Yuan Christian University, Chung Li, Taiwan; 3 Department of Mechanical Engineering, Cheng Shiu University, No.840, Chengcing Rd., Niaosong Dist, Kaohsiung City, Taiwan; 4 Department of Vehicle Engineering, National Pingtung University of Science and Technology, No. 1, Shuefu Road, Neipu, Pingtung 91201 Taiwan

**Keywords:** Kinesio taping, Performance fatigability, Force loss, Supination/pronation

## Abstract

**Background:**

Repetitive exertion in supination/pronation could increase the risk of forearm diseases due to fatigue. Kinesio taping (KT) is a physical therapy technique that decreases muscle tone and musculoskeletal disorders (MSDs) risk. Many assumptions about taping have been made and several studies have considered the taping applications; however, the effect of KT on strength and fatigue of the forearm supination/pronation remains unclear. The purpose of this study was to evaluate the effect of KT on forearm performance fatigability.

**Methods:**

A screwing test was constructed to measure the forearm force loss and screwing efficiency during repetitive supination/pronation. Data from 18 healthy adults who underwent both KT and no taping (NT) sessions were used to investigate the forearm strength change in terms of grip force (GF), driving torque (DT), and push force (PF). The maximal isometric forces before and after the screwing test and force decreasing rate (efficiency) during screwing were evaluated to assess the performance fatigability in KT and NT conditions.

**Results:**

A statistically significant force loss (FL) in maximal isometric GF (*p* = 0.039) and maximal isometric DT (*p* = 0.044); however, no significant difference was observed in maximal isometric PF (*p* = 0.426) between NT and KT. KT provides greater screwing efficiency than NT.

**Conclusions:**

KT could not improve FL in the maximal muscle strength of the forearm in healthy subjects. KT on the forearm was associated with a lesser decline in DT efficiency than NT, implying that KT could decrease the loss rate of muscle strength and delay the development of fatigue; however, the KT did not yield improvements in PF while performing screwing tasks.

## Background

Most upper extremity musculoskeletal disorders (MSDs) are attributed to possible changes in physiological mechanisms and cumulative disorders resulting from manual handling, heavy physical work, uncomfortable postures, repetitive motion, vibration, exposure to prolonged high- or low-intensity loads, or repetitive hand-tool tasks. Several physical therapy techniques have been developed to decrease muscle tone, muscle stress, and performance fatigability (reduction of muscle capacity or power for a given muscle activation) and improve range of motion, proprioception, muscle power, and pain release [1–6].

Performing a screw driving task requires power through hand/finger gripping and repetitive forearm supination/pronation movements to provide the rotational torque. It is a frequent task in orthopaedic surgeons or dentists when performing the orthopaedic traumatic plate/screw fixations or dental implanting [7], manufacturing and processing industries, such as machine/automobile assembly, maintenance/repair activities, gardening, construction work or woodworking tasks [8]. The screwing task includes 3 repetitive motion types: supination/pronation (S/P), wrist extension/flexion (E/F), and radial/ulnar (R/U) deviation [9, 10]. These repetitive movements could increase the risk of upper limb injuries and diseases, and psychophysical studies on repetitive motions of the wrist and hand with maximum frequencies and forces have also been conducted [11–13].

Kinesio taping (KT) is a physical therapy technique commonly used in sports performance enhancement, injury prevention, and rehabilitation for occupational injuries and has gained popularity in recent decades for the treatment of several health conditions. The principles of taping are based on the hypothesis that an external component could aid the function of muscles and other tissues. KT is designed using a thin elastic tape that can be stretched to 40–60% of its original length and can be applied to the skin to provide functional support to the muscle, allowing greater skin traction and power generation, reduction in pain intensity, increase in blood and lymph circulation, which can decrease the risk of muscular fatigue and injury [14–16]. Although the therapeutic benefits of KT in clinical practice have been reported [17–21], several recent studies suggest no significant effects of KT on lower limb function, muscle strength, power, and self-perceived fatigue level in clinical practice [22, 23]. Some studies have addressed the effects of screwing on forearm muscle strength and fatigue [8–10, 12, 13, 24–26]. Based on our literature search, few have examined the effect of KT on the strength and muscle fatigue for forearm supination/pronation activities. Furthermore, the identification of taping and decreased efficiency during screwing remains elusive. Therefore, the objectives of this study are to investigate the effects of KT on forearm performance fatigability in terms of hand gripping force, forearm driving torque (DT) and push force (PF) when performing screwing tasks and to determine the effects of KT on the forearm muscle strength.

## Methods

### Participants

We recruited and enrolled 18 healthy younger adults (15 men, 3 women; mean age, 25.6 ± 7.2 years; range, 20–38 years) participated in our laboratory study. The mean body height and weight were 168.5 ± 13.3 cm and 65.0 ± 16.8 kg, respectively. A power analysis using G Power 3.1.9 was performed to estimate the sample size needed for detecting a within-subject difference between taping and non-taping sessions. With the significance level set at α = 0.05, power at 0.90, and an effect size of 0.8, the power analyses determined that a total of 18 participants was needed. All participants were right-hand dominant and without a history of hand injury. Each participant performed both KT and no taping (NT) sessions. Before participation, each participant was informed about the study details, and they had to sign an informed consent form approved by the local institutional review board (IRB). Characteristics of subjects are listed in Table 1.

**Table 1:** Characteristics of subjects (*n* = 18)

Gender (Female/Male)	3/15
Age (years)	25.6 ± 7.2
High (cm)	168.5 ± 13.3
Body mass (kg)	65.0 ± 16.8
Marital status (married/single)	6/12
Employment (employed/student)	9/9

### Tape application

**Fig. 1:**
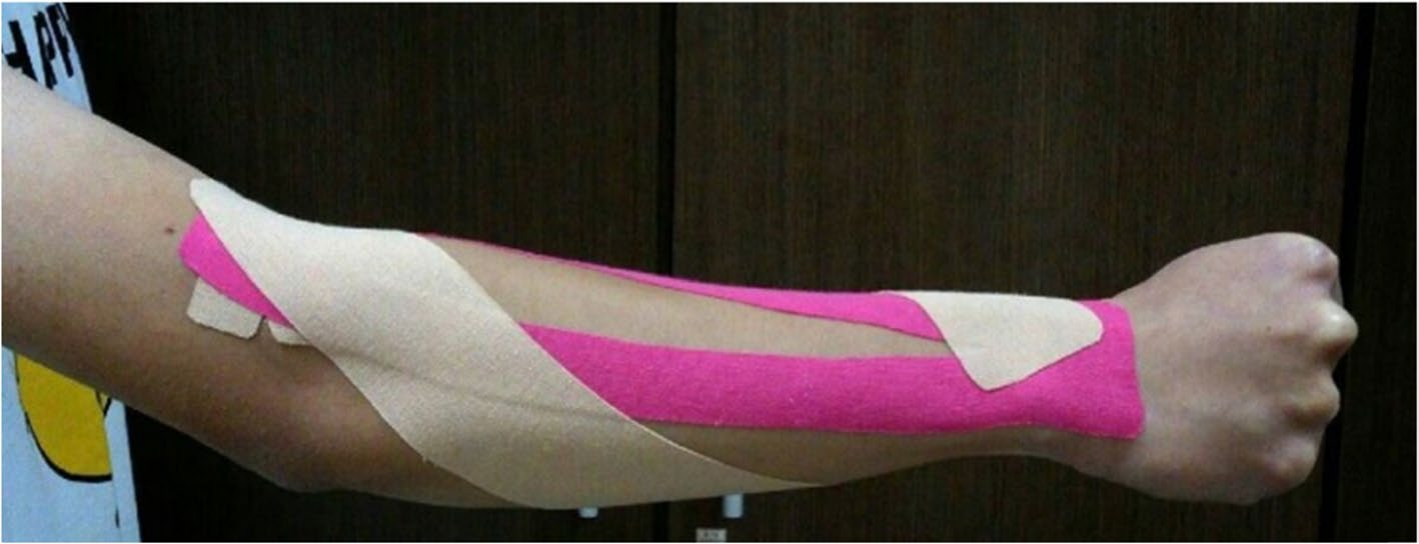
Application of KT to the forearm

Before applying the tape, each participant’s skin was shaved and cleaned with alcohol. The forearm length of the participants was measured by the experimenter. The KT was a standard 5-cm (width) tape (SARASA, PHAROS, Japan) cut in a “Y-strip” and applied with approximately 30% tension over the muscle. The proximal head of the Y-strip was applied to the dorsal side of the wrist, and the tails were placed along the ulnar and radial wrist flexors and extensors to the medial and lateral epicondyle, respectively. Another spiral tape was placed along the forearm (from elbow to the wrist; Fig. 1). To standardize the stretch tension, a 3-cm line was drawn on the tape before application, then the tape was stretched to approximately 4 cm in length for taping (approximately 30% tension). The taping technique was performed by an experienced physical therapist to avoid possible effects on the results.

### Instrumentation

Solid rigid polyurethane foam prosthetic blocks measuring 180 × 65 × 40 mm (Sawbones, Pacific Research Laboratories, Inc., USA) were used as the test media (specimens). The mechanical properties of the prosthetic block satisfied the F-1839-08 standard specifications of the American Society for Testing and Materials. All test blocks (specimens) exhibit constant and uniform material properties with an identical density of 0.64 g/cc. Twenty stainless steel screws (diameter, 3.6 mm; length, 30.5 mm, and pitch, 1.0 mm), commonly used in woodwork or assembled (modular) furniture, were prepared, which were to be inserted into the test block using a 35-mm handle diameter screwdriver to perform the screwing task. Each test block was previously predrilled with twenty 2.0-mm pilot holes (spacing, 25 mm) to guide screw insertion and to prevent the test block from splitting and cracking. A steel plate (30 × 30 × 5 mm) was predrilled with a 3.6-mm screw hole at the centre of the steel plate, and a 3.6 mm screw was inserted; the screw head was welded to the steel plate to prevent relative rotation between the screw and the steel plate. The button side of the plate was smeared with epoxy, and then the screw-plate was inserted at the middle span of the test block until the plate pressed onto the surface of the test block, providing adherence. This fully locked screw-plate test block provided a non-rotatable (fully locked) mechanism to avoid stripping the screw (over-tightening a screw in a hole) and allowed the subject to perform isometric exercises on the fully locked screw. The two ends of the block specimen were then clipped on an experimental platform to perform the screwing tasks. The experimental platform for measuring the driving forces was constructed with two clippers and was installed on a working table for performing screwing tasks. The height of the working table was individually adjusted based on the participant’s stature.

**Fig. 2:**
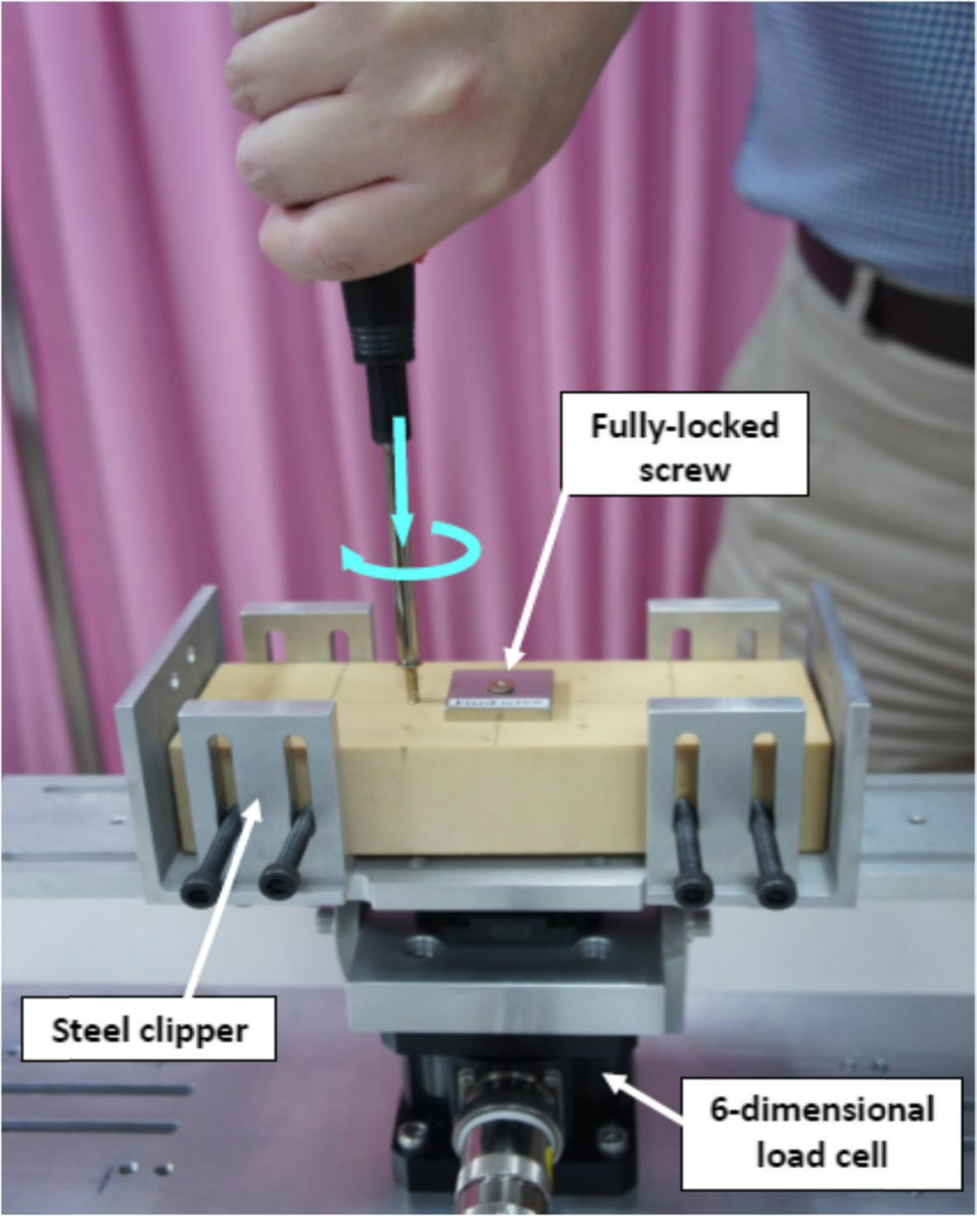
The experimental setup for screwing task

A digital handgrip dynamometer (Jamar Plus+ Digital Hand Dynamometer, Sammons Preston, Bolingbrook, IL, USA) was used to measure the maximal grip force (GF) before and after the screwing tasks. A six-dimensional load cell (MC3A AMTI, Watertown, MA, USA) was installed under the test blocks (prosthetic blocks) to measure the maximal DT and sub-cycling forces during screwing. The measured force level was digitized by analogue-to-digital conversion at 100 Hz (16-bit resolution) and stored on a computer for further analysis. The overall experimental setup is shown in Fig. 2.

### Experimental protocol and measurements

**Fig. 3:**
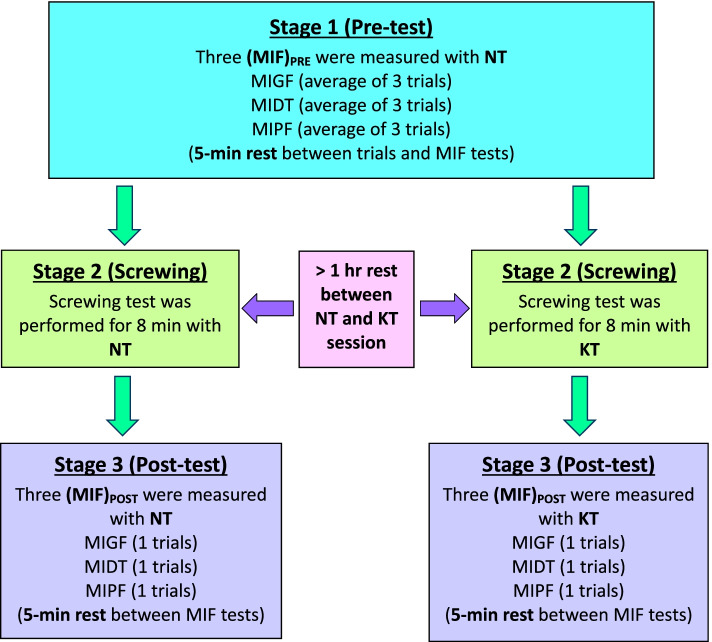
Overall view of the experimental procedures and measurements in this study

The experiment was conducted in three stages. The participants were asked to be in the laboratory on the same day and to complete the screwing tasks under both KT and NT conditions. Figure 3 shows an overview of the experimental procedures and measurements. The experimental procedures and details of the three stages are described in the following section:

- Stage 1: Pre-fatigue MIF measurement with NT

To perform the screwing task, the exertion force from the forearm includes the GF, DT, and PF. In this stage, each participant was required to perform three maximal isometric force (MIF) tests under NT conditions, including the maximal isometric gripping force (MIGF), maximal isometric driving torque (MIDT), and maximal isometric push force (MIPF) with 3-trials for each. A 5-min rest period between trials was provided to minimize potential muscle fatigue. The 5-min rest period has shown to be an acceptable rest period after a localized muscle fatiguing contraction. For each MIF test, the average value of the three trials was used to determine the participant’s pre-fatigue MIF values [(MIF)_PRE_]. This value was considered as the initial maximal strength and was used to normalize the applied forces measured in stages 2 and 3. The measurement methods and data processing for MIGF, MIDT, and MIPF, are as follows:

1. *MIGF*

Participants were asked to sit on a chair without leaning, with the shoulder in slight abduction (about 15°), the elbows in about 90° flexion, and forearms neutral in supination/pronation. An electronic handgrip dynamometer (Jamar Plus+ Digital Hand Dynamometer, Sammons Preston, USA) was used to measure the maximal GF values before and after the screwing tasks. A 3-trial test was performed with a 5-min rest between trials. In the first stage, the average of the maximal measured values from 3 trials was recorded as the (MIGF)_PRE_ in the pre-fatigue condition.

(2) *MIDT*

After completing the MIGF measurements, a 5-min rest was provided. This was followed by three trials of the MIDT tasks on the head of the fixed screw (at the middle span of the specimen) to obtain pre-fatigue MIDT [(MIDT)_PRE_]. As described in section 2.3, the locked screw was welded to a square steel plate and fixed on the wood specimen, providing the non-rotatable mechanics, for performing the isometric testing. The participants used a screwdriver to exert the driving torque from zero to maximum in 3 s and maintain the maximal force for approximately 5 s and then release to complete the trial of a measurement. The steady-state part (holding period) of the measured values during the 1 s time window was used as the mean driving torque of the trial. This could eliminate the influence of the start of the plateau contraction and the ramp-up to MIF. At this stage, the mean driving torque values from each trial were averaged and used as the MIDT before screwing [(MIDT)_*PRE*_].

(3) *MIPF*

The measurement method for MIPF was similar to that for MIDT. After completing the MIDT test and after a 5-min break, MIPF was assessed over three trials to obtain the pre-fatigue value. Participants practised their maximal push force on the head of the fixed screw and maintained it for approximately 3 s. During isometric pushing, force generation data were measured and recorded. A 1-s time window for the steady part of the measured values was used for averaging an MIPF trial test. Pre-fatigue MIPF [(MIPF)_*PRE*_] was evaluated by averaging the three trials values.

- Stage 2: Performing and measuring the screwing task with NT and KT

**Fig. 4:**
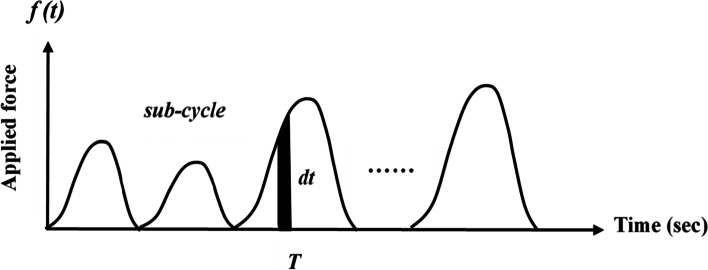
Schematic curve of repeated sub-cycle with time during screwing

After the first stage, a 5-min break was provided to each participant. Subsequently, the KT and NT sessions were individually applied in stages 2 and 3. The participant was randomly assigned (determined by tossing a coin) to begin the second stage with or without taping. Each participant was required to perform the screwing task for 8 min under NT and KT conditions. A 1-h rest was provided between the NT and KT sessions. The applied DT and PF were continuously recorded to obtain the sub-cycle force responses. Generally, muscle fatigue can be identified as the gradual decrease in the force generation capacity of a muscle or the duration in which a given level of maximum voluntary contraction (MVC) can be sustained; it can be measured as a reduction in muscle force or as the exhaustion of contractile function [27]. Figure 4 shows a schematic of the repetitive exertion (sub-cycle) when performing the screwing task. During the screwing test, the task was performed in a repetitive hand-arm movement with a dynamic force generation activity, and the muscle contraction was not sustained constantly; quantifying the degree of muscle strength and fatigue was therefore difficult.

In this study, the impulse produced during the sub-cycle was defined as the integral of the applied force, *f*(*t*), over the time interval, *dt*. During cycling, each minute of the repetitive exertion can be analogized to an average force generated across 1-min intervals; therefore, the averaged applied force at *j-th* minute interval can be expressed as:

1 $${\overline{F}}_j=\frac{\int_{T_{j-1}}^{T_j}f(t) dt}{\int_{T_{j-1}}^{T_j} dt}$$

Where:

*f* (*t*): applied force (measured driving torque or push force) at time *t.*

*T*_*j*_: duration of force applied (sub-cycle) until *j-th* min (in this study, *j* = 1 to 8).

To evaluate the force decline during screwing, the applied force in 5 s from the beginning was averaged as the initial screwing force (${\overline{F}}_i$). Thus, the percentage of force decline (or screwing efficiency, SE %) from the first to all other minutes can be normalized with the initial screwing force and expressed as:

2 $${SE}_j\ \left(\%\right)=\left(\frac{{\overline{F}}_j}{{\overline{F}}_i}\right)\times 100$$

In practice, the applied forces measured in a screwing task include the combination of DT and PF. Therefore, the applied force at the *j-th* minute [${\overline{F}}_j$ in Eqs. (1) and (2)] should be expressed as driving torque (*DT*_*j*_) or push force (*PF*_*j*_), and the ${\overline{F}}_i$ should be replaced by (*DT*_*i*_) or (*PF*_*i*_).

- Stage 3: Post-fatigue MIF measurement with NT and KT

After the screwing task (stage 2), stage 3 was performed immediately. A single-trial test each for measuring MIGF, MIDT, and MIPF was performed to obtain post-fatigue maximal isometric force values [(MIF)_POST_]. No rest time was provided between stages 2 and 3 to avoid recovery between stages. The measurement process at this stage was similar to that for the first stage; however, only the single-trial task was performed. The single-trial measurement was to avoid the effect of muscle recovery between trials and fatigue due to trial times. If one of the taping sessions (NT or KT) was finished from stage 3, then the other taping session was conducted from stage 2. More than 1 h of rest was required between KT and NT sessions to avoid the accumulation of testing effects or muscle fatigue [28, 29]. Because the pre-fatigue maximal isometric forces [(MIFs)_POST_] were received from stage 1, and the post-fatigue maximal isometric forces [(MIFs)_POST_] were measured from this stage, the FL before and after the screwing task can be calculated using the formula FL = (MIF)_PRE_ – (MIF)_POST_. The FL was regarded as the degree of muscle fatigue caused by driving the screws [30]. The percentage of FL for each participant can be normalized by their pre-fatigue condition (MIF)_PRE_ as follows:

3 $$\mathrm{FL}\ \left(\%\right)=\left(\frac{{\left(\mathrm{MIF}\right)}_{\mathrm{PRE}}\ {\left(\mathrm{MIF}\right)}_{\mathrm{POST}}}{{\mathrm{MIF}}_{\mathrm{PRE}}}\right)\times 100\%$$

Where (MIF)_PRE_ and (MIF)_POST_ correspond to the MIFs measured at the pre-test and post-test conditions, respectively.

### Statistical analyses

We used the MIFs data (MIGF, MIDT, MIPF), obtained from the pre-test and post-test conditions, and driving force (GF, DT, and PF) received during the screwing test for statistical analysis. All results are presented as mean and standard deviation (mean ± SD). The independent t-test was performed to determine the contribution of taping effects. Paired t-test was used as a post-test to compare the MIFs before and after the screwing test. One-way analysis of variance (ANOVA) helped to examine whether the participants’ generated force (screwing force) differed during every 1-min interval. Bonferroni post hoc tests were used to determine individual significant differences. All analyses were performed using statistical package software (SPSS 13.0 Inc., Chicago Ill, USA), with significance considered at *p* ≤ 0.05.

## Results

Performance fatigability can be defined as the magnitude or rate of change in a performance criterion relative to a reference value over a given time of task performance or measure of mechanical output. This study assessed the change in the MIFs before and after performing a screwing task. Table 2 presents the effects of NT and KT on the MIFs. Significant differences were observed in MIGF and MIDT between the NT and KT sessions (MIGF: *p* = 0.039; MIDT: *p* = 0.044); however, no significance in MIPF with NT and KT (*p* = 0.426). Table 3 presents the force loss (FL) in MIGF, MIDT and MIPF. Experimental results show in the NT condition, the percentages of FL in MIGF, MIDT, and MIPF were 31.0, 24.2, and 26.5%, respectively. In the KT condition, the percentages of FL in MIGF, MIDT, and MIPF were 27.4, 19.5, and 25.7%, respectively, indicating significant FL after screwing (*p* ≤ 0.001 for every MIF). The results indicate that KT does not seem likely to improve forearm strength loss.

**Table 2:** Comparison of post-fatigue MIFs with NT and KT

Taping	MIGF (N)	MIDT (N-m)	MIPF (N)
**NT**	280.8 ± 36.0	4.0 ± 0.6	99.9 ± 6.6
**KT**	293.3 ± 43.3	4.2 ± 0.7	100.4 ± 9.1
***p*-value**	0.039 *	0.044 *	0.426

^*^Significant difference between NT and KT conditions. Data expressed as mean ± SD

**Table 3:** Force loss in MIGF, MIDT and MIPF

MIFs	Taping	Pre-fatigue	Post-fatigue	FL (%)	***p***-value
**MIGF (N)**	NT	408.7 ± 60.9	280.8 ± 36.0	31.0 ± 5.3	< 0.001♦
	KT		293.3 ± 43.3	27.4 ± 3.0	< 0.001♦
**MIDT (N-m)**	NT	5.2 ± 0.7	4.0 ± 0.6	24.2 ± 5.7	< 0.001♦
	KT		4.2 ± 0.7	19.5 ± 5.1	< 0.001♦
**MIPF (N)**	NT	135.2 ± 8.2	99.9 ± 6.6	26.5 ± 3.4	= 0.001♦
	KT		100.4 ± 9.1	25.7 ± 5.8	< 0.001♦

^♦^Significant difference in FL (%). Data expressed as mean ± SD

**Fig. 5:**
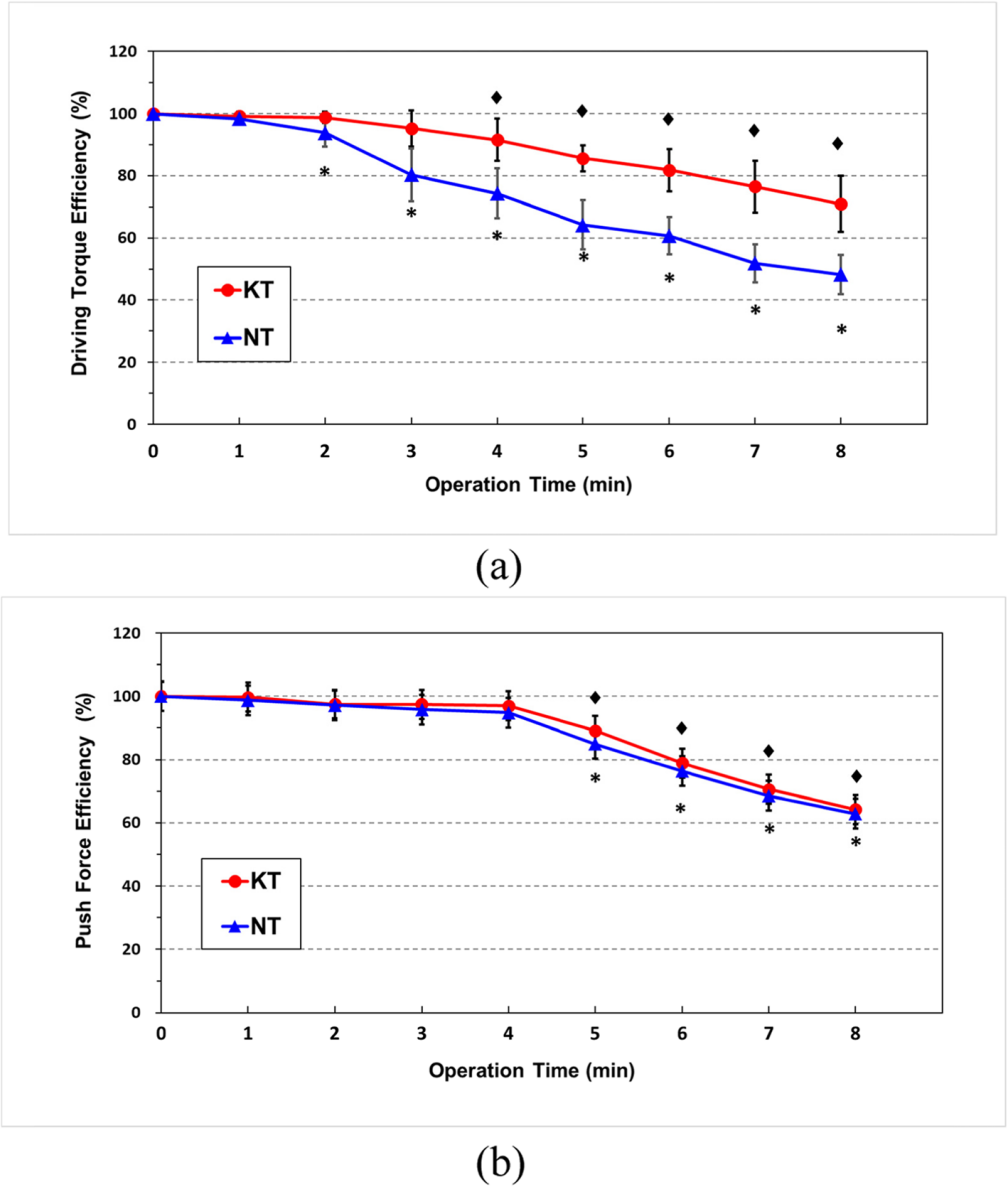
Screwing efficiency-task duration relations: **a** Driving torque (**b**) Push force. (**♦**) significant difference compared with the first minute in KT. (*) significant difference compared with the first minute in NT

Figure 5 plotted the screwing efficiency-task duration relations. The screwing efficiency was evaluated by Eq. (2). Figure 5(a) showed that the values of DT efficiency for the participants with KT were higher than those for participants without taping (NT). The efficiency curve decreased significantly from 2 min for participants with NT but no statistical difference was found until 4 min for the KT session. There was a significant PF efficiency reduction at 5 min for both NT and KT sessions [Fig. 5(b)].

## Discussion

KT is being increasingly used in clinical practice, and several studies have reported KT as a therapeutic intervention that may improve outcomes with lower costs. Some studies have reported that KT application may improve muscle strength and motor function and recommend the use of KT as a treatment resource and even as a tool to assist in injury prevention [20, 28, 31–39]. These studies presented that the stretch applied on the tape provides a pre-pulling force on the skin, stimulating cutaneous mechanoreceptors and altering motor neuron activation, thereby improving communication with mechanoreceptors and increasing the number of motor units recruited. However, similar studies have reported this stimulus was inadequate to increase muscle strength. The taping method is based on the hypothesis that an external component could help improve the function of muscle and other tissues. Different taping techniques with varying stretches can provide different tactile stimuli. Decreasing the taping tension may help in reducing the tactile stimulus and the recruitment of motor units promoted by KT [17, 19, 40–45]. Different theories have attempted to explain the mechanisms underlying the increased neuromuscular recruitment with KT, including the facilitation of neuromuscular stimulus, activation of cutaneous receptors provided by the tactile stimulus, increased blood and lymph circulation, and a reduction in inflammation and pain, improving the contraction ability of the damaged muscle [32, 42]. However, these properties are not sufficient to improve the factors assessed in the previous studies.

A gold standard to determine the true incidence or prevalence of occupational cumulative traumatic forearm disorders is lacking. Several studies used the time to failure to assess muscle fatigue while participants sustained an isometric contraction [46, 47]. In this study, the loss of the maximal exertion before and after the screwing task was evaluated and used as the index to quantify muscle performance fatigability. However, during screwing, the test was performed dynamically; determining the cumulative trauma and rigorously defining and quantifying muscle fatigue under dynamic or repetitive motion is challenging. Moreover, the participants performed the screwing task at their own pace, operating process, and posture based on personal physiological conditions. This could have spontaneously decreased their exerting force and extended their operating time to avoid excessive local muscle fatigue. For dynamic tasks, frequent shorter rest periods amounting to the same total rest durations are better than occasional longer rests for preventing accumulated muscle fatigue [48]. For the same total rest duration, the frequent short recovery time during the working cycle could effectively reduce muscle fatigue than using breaks or recesses between prolonged working periods. To quantify the repetitive sub-cycle load, we used impulse and averaging to equalize the dynamic (repetitive) force to an averaged static force. Thus, work efficiency can be used as the index for the assessment of muscle fatigue. The screwing efficiency (Eq. 2) was expressed as the applied force per unit time (force/time), which can be used to objectively compare the level of exertion during work or exercise.

With regard to the stretch applied to the tape, Chang et al. [17] indicated that there no statistically significant improvements were observed in maximal grip strength when KT was applied. In their study, KT was applied over the common wrist flexor muscles from insertion to origin with 15 to 25% tension in healthy participants. However, Resti et al. [49] showed that KT can improve power or grip strength and active range of motion of the wrist and thumb with 30% of the tape in the muscular zone. In our study, the results did not show significant benefits on FL; we applied the tape in the direction of muscle origin to insertion with 30% stretch. The pre-stretch could be insufficient to extend the forearm endurance capacity. Therefore, further study is needed regarding the performance fatigue driving effect of KT tension in the forearm.

During high-intensity submaximal exercise, muscle fatigue and decreased efficiency are intertwined closely [50]; therefore, muscle fatigue can be related to a decreased efficiency of muscle contraction. In this study, screwing efficiency was used to quantitatively estimate the degree of muscle fatigue during cyclic exertion. Similar to the power rate, the average applied force was calculated as the force generated per minute. The “efficiency” implies the change of power rate during the working or exertion period. A higher efficiency or a high-power rate indicates a greater “force” or “work” generated in a short period, which implies providing a high working efficiency during exercise. In contrast, a low-power rate results in poor working efficiency. Figure 5(a) shows the values of efficiency significantly decreased at 2 and 4 mins for NT and KT, respectively. This implies that KT could maintain the forearm DT endurance for 4 min; however, only 2 min could be achieved without taping. Figure 5(b) shows that the efficiency of PF significantly declined from 5 min in both NT and KT. We hypothesize that in our driving task, PF was generated mainly from the flexion strength of the elbow, however, the biceps mainly contribute to elbow flexion. In this study, the tape was not applied on the biceps (only on the forearm); hence, the benefit of KT on PF was not obvious. Based on the results of efficiency (Fig. 5), we think that the NT could be more fatigable than KT when performing forearm supination/pronation.

This study has several limitations. First, this study only observed the immediate effects of taping, the effects of longer tape application could yield different results. Although we did not find any significant improvement in muscle strength and motor function in the present investigation, further studies are needed to ascertain the various possibilities of KT application in a wide range of occupational tasks and populations. Second, the taping was applied on the main muscles but not applied on the muscles or tendons apart from the main ones, which could have resulted in insufficient improvements. Third, the placebo taping or a different pre-stretch degree of taping was not used in the current study; hence, sensory stimulation on the skin would have differed between NT and KT conditions. Fourth, the application of KT was used to complement treatment such as in post-injury and orthopaedic patients and athletes. We studied the effects of taping in healthy individuals without any forearm-related diseases, which could be the reason for no apparent efficiency gain with KT. Fifth, the sample size is small, the findings could not be generalizable, which may lead to a negative impact on the validation of the statistical tests. Finally, the results are limited to the upper-arm posture at elevation and elbow flexion to push down the screw insertion; muscle stress and fatigue could differ with other arm postures such as horizontal screwing or upward insertion, and taping in such cases could lead to different results.

## Conclusion

The application of KT could not improve FL in the maximal muscle strength of the forearm in healthy subjects. KT on the forearm was associated with a lesser decline in DT efficiency than NT, implying that KT could decrease the loss of muscle strength and delay the development of fatigue; however, did not yield improvements in PF performance while performing screwing tasks. The outcomes of KT rely on the taping technique, mechanical behaviour of the tape material, clinical experience and duration of taping, more rigorous studies with a larger sample size are needed to quantitatively determine the effects of taping stretch on the forearm muscle performance fatigability.

## Data Availability

All data from the study are presented in the manuscript.

## Abbreviations

KT: Kinesio taping
NT: No taping
MSDs: Musculoskeletal disorders
S/P: Supination/pronation
E/F: Extension/flexion
R/U: Radial/ulnar
GF: Grip force
DT: Driving torque
PF: Push force
FL: Force loss
MIF: Maximal isometric force
MIGF: Maximal isometric gripping force
MIDT: Maximal isometric driving torque
MIPF: Maximal isometric push force
MVC: Maximum voluntary contraction
SE: Screwing efficiency
